# Design and key construction technology of steel-concrete-steel sandwich composite pylon for a large span cable-stayed bridge

**DOI:** 10.1038/s41598-023-33316-7

**Published:** 2023-04-24

**Authors:** Bida Pei, Aixiu Chong, Huan Xia, Xueyun Kang

**Affiliations:** 1 College of Civil Engineering, Changsha University of Science & Technology, Changsha, Hunan China; 2 CCCC Second Harbor Engineering Company Ltd., Wuhan, Hubei China

**Keywords:** Engineering, Civil engineering

## Abstract

This paper introduces a new type of steel-concrete composite pylon that has been applied to Nanjing Fifth Yangtze River Bridge (a three-pylon cable-stayed bridge with a main span of 600 m). For this new type of pylon, the steel shells are connected with concrete through PBL shear connectors and studs, and the inner steel shells are connected with the outer steel shells by angle steels. Numerical analysis and full-scale model tests show that the pylon structure exhibits excellent mechanical properties and construction performance. The application of BIM technology, the research and development of special spreaders and construction platforms ensure the precise installation of structures. Highly factory-manufactured modular assembly of the reinforced steel shell structure can effectively reduce the intensity and difficulty of on-site operations, and improve the quality of the project, with low construction risks. Whereas, the successful application of this steel-concrete-steel sandwich composite pylon marks the formation of a complete set of construction technology of steel-concrete-steel sandwich composite pylon, which can be widely used in similar bridges.

## Introduction

Pylons are the critical load-bearing components of cable-supported bridges responsible for transmitting the loads from the cables to the bridge foundation. The stability of the bridges is therefore dependent on the stability and stiffness of the pylons. Research and development of a pylon structure with improved mechanical properties, industrial prefabrication, faster erection, and reliable quality are paramount for bridge engineering.

Traditionally pylons of cable-supported bridges are made using steel structure pylons or concrete structure pylons^1^. Although the steel structure pylons have the advantages of factory prefabrication and modular erections, its application is far less than that of concrete pylons due to the high cost. Due to the high stiffness requirements for the pylon, the pylons must have a larger cross-sectional area, and thus more steel is used, resulting in a construction cost of about three times that of a concrete pylon. Concrete pylon has the advantages of high rigidity and good stability and lower construction costs. Still, its construction method is a series of complicated stages, i.e., rigid skeleton installation, rebar binding, formwork installation and adjustment, and concrete pouring. The construction operations mainly rely on manual labour with low standardization and prefabrication, which results in long construction periods, high site operation intensity, high risk, and extended equipment occupancy cycles.

The steel-concrete composite pylons exhibit many advantages over the steel and concrete pylons. The steel structure can save much time through factory prefabrication and modular installation. At the same time, it can also be used as a template for concrete pouring. The steel structure constrains the concrete, further improving its bearing capacity. The combination of steel and concrete also inherits the advantages of the high rigidities of the concrete pylon.

Steel-concrete composite pylons are primarily used in pylons with complex geometric shapes. For instance, due to the complex geometry of the Alamillo cable-stayed bridge, the original design of the reinforced concrete pylon was changed to a composite structure in order to reduce the time required for construction. Therefore, an outer metal case connected to the concrete replaced many steel bars, which would have taken far longer to install^2^. The composite action is achieved by stud connectors directly welded in the main steel plates forming the outer case and the horizontal stiffeners of the main plates that were also accounted for in the transmission of the shear force between the steel and concrete. Hsu et al. researched the observed behaviour of sandwich box columns which consisted of double thin-walled steel tubes with concrete between them subjected to combined bending and axial loading. The results show that the strong performance of the sandwich members is higher than their corresponding concrete-filled tubes member. The improvement in strength reached up to 45 per cent for sandwich sections with non-compact outer tubes^3^. The upper pylon column of Stonecutters Bridge adopts a steel-concrete composite structure. The steel structure is made of stainless steel, and only welded studs are used to connect the steel and the concrete^6^. Tao et al. studied the strength and rigidity of concrete-filled steel tubular stub columns with inner or outer welded longitudinal stiffeners under axial compression^4^. Xie et al. studied an innovative form of steel-concrete-steel sandwich construction, in which the two steel plates are interconnected by a series of transverse bar connectors simultaneously friction welded at both ends^5^. Zeng et al. designed and produced five specimens with perforated plate connectors and five specimens with welded stud connectors to study the observed behaviour of double-skin steel-concrete composite pylon under axial load and combined constant axial load with cyclic lateral load, respectively^7^. Leng W.H. studied the calculation method of the PBL shear connectors bearing capacity of the prestressed steel-concrete composite curve pylon of the Lichuan Bridge and the factors affecting the shrinkage and creep of the concrete in the steel shell^8^. J.Y. Richard Liew et al. researched the performance of an innovative sandwich composite structure with J-hook connectors, including sandwich composite beams, sandwich composite plates, and composite sandwich walls subject to blast, impact, fatigue, and static loads^9^. Wei et al. studied on the force transmission mechanism of a steel-concrete composite pylon with an upper steel pylon and a lower concrete pylon at the junction of steel and concrete by a scaled-scale model test^10^. Wang et al. studied the effects of different cross-section forms and shear connectors on the steel-concrete composite pylon, and the results showed that, compared with the rectangular cross-section, the rectangular cross-section with chamfers has a higher ability to resist local buckling, and the shear connectors can greatly increase the bearing capacity and ductility of the steel-concrete composite pylon^1^.

However, steel-concrete composite pylons are mostly used in cable-stayed bridges with spans less than $200\,\textrm{m}$ in China, but such pylons usually only have an outer steel shell. The publicly reported application cases of steel-concrete composite bridge pylon projects are shown in Table 1. This study introduces a new type of steel-concrete composite pylon, the steel shell is connected to concrete through PBL shear connectors and studs, and the inner steel shell is connected to the outer steel shell by angle steels. The penetrating rebars of the PBL shear connectors are also used as the primary reinforcement of the concrete. The inner and outer steel shells are also the formwork for concrete pouring and, finally, form a steel-concrete-steel sandwich composite pylon. This type of pylon is, for the first time, applied to cable-supported bridges with a span of more than $500\,\textrm{m}$. This type of pylon has the advantages of high prefabrication, rapid construction, reliable quality, good toughness and plasticity, and a good appearance. This article will specifically introduce the design and construction of a steel-concrete-steel sandwich composite pylon applied in a cable-stayed bridge with a main span of $600\,\textrm{m}$. The research of this project is of great significance to promote the industrial upgrade of bridge construction from construction to manufacturing, and it could also be used as a reference for similar bridge types.

**Table 1:** Engineering application of steel-concrete composite bridge pylon(Incomplete Statistics).

Name	Type	Span arrangement/m	Site	Year built	Vertical pylon height/m	Bridge pylon form
Alamiro Bridgen	Single-pylon cable-stayed bridge without back ropes	200	Seville,Spain	1992	134.25	Inclined single column
Tsurumi Navigation Bridge	Cable-stayed bridge	510	Yokohama, Japan	1994	180	Strong skeleton reinforced concrete
Normandy Bridge	Cable-stayed bridge	856	Normandy, France	1995	215	Steel anchor box and partial reinforced concrete
Si-dong Bridge	Double-pylon cable-stayed bridge	69+140+69	Nanhai, Guangdong	1996	36	Single column(round)
Ting Kau Bridge	Three-pylon cable-stayed bridge	448+475	Hong Kong, China	1998	194	Single column (rectangular)
Wanan Bridge	Cable-stayed bridge	72+140+72	Wanzhou, Chongqing	2001	36	Single column(round)
Changsan Road Bridge	Single pylon cable-stayed bridge	80+88	Huabei, Anhui	2001	50	Portal(Round)
Rongho Bridge	Single pylon cable-stayed bridge	41.2+33.8+145	Wuxi, Jiangsu	2003	74.3	Single column(round)
Nanjing Yangtze River Three Bridges	Double pylons with steel pylons and steel box girder cable-stayed bridge	63+257+648+257+63	Nanjing, Jiangsu	2005	215	Herringbone (arc-shaped)
Houhu Bridge	Solo Pylon Cable-stayed Bridge	34+56+128	Wuhan, Hubei	2008	67	Solitary column(round end shape)
Gokan Bridge	Self-anchored single-pylon suspension bridge	48+180+180+48	Shenyang, Liaoning	2012	67	Solitary column(round end shape)
Liujiaxia Bridge	Double Pylon Suspension Bridge	536	Linxia,Gansu	2014	74.81	Portal (circular)
Xiongzhou Bridge	Single pylon cable-stayed bridge	100+85	Nanjing, Jiangsu	2016	66(above bridge deck)	Arch pylon
Shuangye Island Bridge	Twin-pylon cable-stayed bridge	36+66+66+36	Zhangzhou, Fujian	2016	90	A-shaped
Icheon Bridge	Backless cable-stayed bridge	51.5+138+55	Dongguan, Guangdong	2017	68.5	Inclined double column type curved pylon
Lianchi Street Overpass	Double-pylon cable-stayed bridge	76+150+76	Hebei Xingtai	2018	29.793	Dumbbell-shaped double pylon
Changzheng Bridge	Single-pylon cable-stayed bridge	80+100	Lu’an, Anhui	2018	59.2	Inclined single column(round)
Dongfeng Road Interchange	Single pylon cable-stayed bridge	45+45+169	Siping, Jilin	2019	75	Solitary column
Nanjing Yangtze River Five Bridges	Three-pylon cable-stayed bridge	80+218+600+600+218+80	Nanjing, Jiangsu	2020	175.4	Para-bridged diamond-shaped pylon column
Bahe Yuanshuo Bridge	Self-laying twin-pylon suspension bridge	50+116+300+116+50	Xi’an, Shaanxi	2021	123	Parabrachially oriented double limb
Binhai Bay Bridge	Single-pylon cable-stayed bridge	60+200+200+60	Dongguan, Guangdong	2022	149.8	Single column

## Bridge description

**Figure 1:**
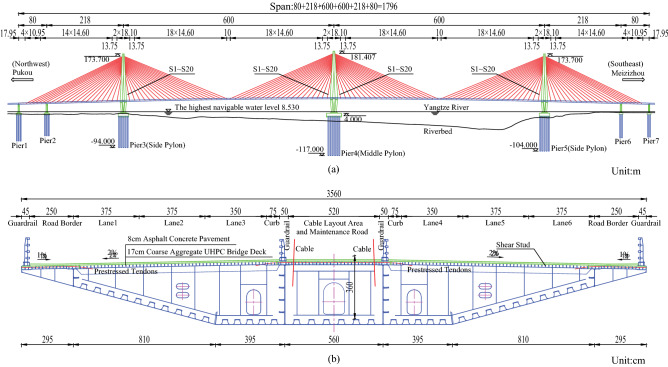
Overview of the Jiangxinzhou Yangtze River Bridge in Nanjing. (**a**) elevation view; (**b**) section of steel boxed girder.

The bridge in this study is the Jiangxinzhou Yangtze River Bridge in Nanjing (JYRB), known as the Fifth Nanjing Yangtze River Bridge, which was opened in 2020. JYRB is a steel-UHPC composite girder cable-stayed bridge with three pylons and double cable planes. Its span arrangement is $80\,\textrm{m}+218\,\textrm{m}+2\times 600\,\textrm{m}+218\,\textrm{m}+80\,\textrm{m}$. Figure 1 shows the layout of JYRB. The main girder, with a height of $3.6\,\textrm{m}$ and a width of $35.3\,\textrm{m}$, includes six traffic lanes and two footpaths. Moreover, it comprises a flat steel box girder and a UHPC layer with coarse aggregates connected through shear studs. The three pylons are one middle pylon with a height of $177.407\,\textrm{m}$ and two identical side pylons with a height of $169.7\,\textrm{m}$. The three main pylons are all diamond-shaped pylons in the longitudinal direction but single-pillar pylons in the transverse direction. Twenty pairs of cables are anchored on each side of the pylons, and a total of 240 cables are used in this bridge.

**Figure 2:**
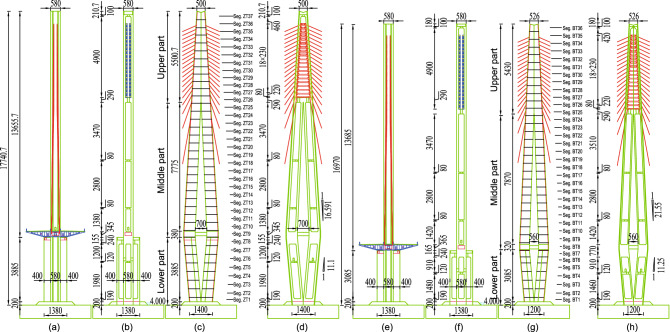
Middle pylon and side pylon structure layout. (**a**) middle pylon in the transverse direction layout; (**b**) middle pylon in the transverse direction profile layout. (**c**) middle pylon in the longitudinal direction layout; (**d**) middle pylon in the longitudinal direction profile layout. (**e**) side pylon in the transverse direction layout; (**f**) side pylon in the transverse direction profile layout; (**g**) side pylon in the longitudinal direction layout; (**h**) side pylon in the longitudinal direction profile layout. (Unit:cm).

**Figure 3:**
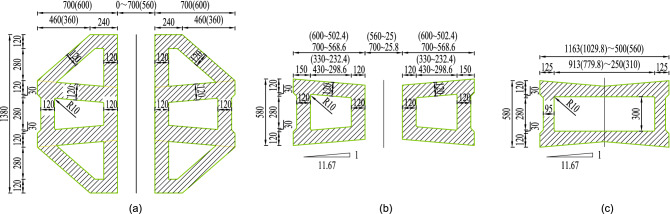
Sections of the pylon: (**a**) standard section of lower part of the pylon; (**b**) standard section of middle part of the pylon; (**c**) standard section of upper part of the pylon. (Unit:cm).

**Figure 4:**
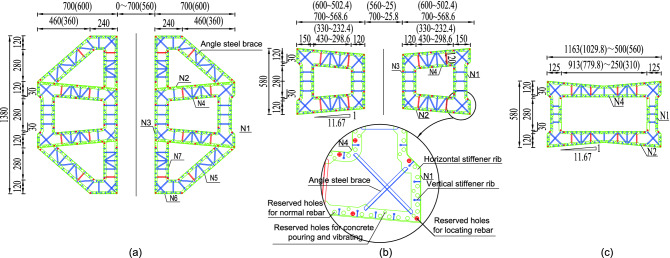
Steel shell structures of the pylon: (**a**) steel shell structure of lower part of the pylon; (**b**) steel shell structure of middle part of the pylon; (**c**) steel shell structure of upper part of the pylon. (Unit:cm).

With the middle pylon as an example, the pylon can be divided into the upper, middle, and lower parts, and a cross beam is designed at the junction of the middle and lower parts (see Fig. 2). The height of the upper, middle and lower parts are $55.007\,\textrm{m}$, $81.550\,\textrm{m}$, and $40.850\,\textrm{m}$, respectively. The lower part is a longitudinal double-limbed structure, and each limb adopts the box section with three cells (see Fig 3a).The two limbs of the lower part separate upward gradually, and the maximum distance between the two limbs is $7\,\textrm{m}$, while the two limbs of the middle part gather upward gradually until the upper part merges into one limb. The standard sections of the middle and upper parts are shown respectively in Fig. 3b,c. The steel shell structures are shown in Fig. 4 for those pylon sections.

The middle(side) pylon is divided into 37(36) segments, and the height of the standard(highest) segment of the pylon is 4.8$\,\textrm{m}$($5.2\,\textrm{m}$). The steel shell of the pylon is composed of inner and outer steel panels, vertical and horizontal stiffeners, shear studs, connecting angle steels, etc (Fig. 2). The standard thickness of the outer steel plate is $14\,\textrm{mm}$, of which $20\,\textrm{mm}$ and $16\,\textrm{mm}$ are used for part of the outer steel plate above and below the lower beam and the first segment of the lower part pylon. The standard thickness of the inner wall plates is $6\,\textrm{mm}$. The vertical stiffener size is $128\times 10\,\textrm{mm}$, while the horizontal stiffener size is $200\times 10\,\textrm{mm}$. The standard spacing of those stiffeners is $400\,\textrm{mm}$, the stiffeners are locally widened to suit the angle steel connection. The $\phi 60\,\textrm{mm}$ holes are set on the vertical stiffener to pass through the horizontal rebars, and the $\phi 86\,\textrm{mm}$ and $\phi 80\,\textrm{mm}$ holes are set on the horizontal stiffener to pass through the vertical rebars. In addition, some $\phi 70\,\textrm{mm}$ holes are set on the horizontal stiffeners for concrete pouring and vibrating. Rebars adopt HRB400 grade rebars, and the diameters of vertical rebars and horizontal rebars are $36\,\textrm{mm}$ and $22\,\textrm{mm}$ respectively. It is worth noting that because the rebars pass through the stiffeners, the stiffeners and the rebars are no longer just stiffeners and rebars in the simple sense. They combine to form PBL shear connections, thereby realizing the collaborative work of the steel structure and the concrete. The shear studs with a diameter of $22\,\textrm{mm}$ and a height of $150\,\textrm{mm}$ after welding are welded to the center of the rectangular grid formed by the vertical stiffener and the horizontal stiffener, which further strengthens the connection between the concrete and the steel shell. The outer and inner steel plates are connected into a whole through the $L75\times 8\,\textrm{mm}$ angle steel, which is beneficial to control the deformation of the steel shell when the concrete is poured and increases the steel shell’s overall stiffness segment. The inner and outer steel panels and stiffeners of the steel shell are made of Q345C steel, and the rest of the panels are made of Q235B steel. The concrete in the pylon is C50 compensated shrinkage concrete.

The crossbeam is a rectangular steel box girder with a height of $2.0\,\textrm{m}$ and a width of $4.6\,\textrm{m}$. The steel plate thickness of the crossbeam is $20\,\textrm{mm}$. The height of the longitudinal stiffener is $160\,\textrm{mm}$, the thickness of the plate is $16\,\textrm{mm}$, and the thickness of the diaphragm is $12\,\textrm{mm}$. There are 12 bundles of $\phi 15.2-22$ high-strength low-relaxation external prestressed steel strands being arranged along the lower beam, which are tensioned at both ends with a tension control stress of $1209\,\textrm{MPa}$. In addition, there are 12 bundles of $\phi 15.2{-}15$ high-strength low- relaxation external prestressed steel strands are arranged along the longitudinal direction at the upper part and middle part junction, which are tensioned at both ends with a tension control stress of $1395\,\textrm{MPa}$.

## Design of the pylons

## The idea of the pylons

Unlike traditional cable-stayed bridges, the three-pylon cable-stayed bridges’ global stiffness is usually more negligible due to the two middle spans without auxiliary piers. Therefore, different methods are considered to improve the global stiffness of the three-pylon or multi-pylon cable-stayed bridge structure and the stability of the middle pylon:

1. Improve the pylon’s stiffness to ensure the structure’s global stiffness and structural performance;
2. Increase the height of the main girder to improve its stiffness of the main girder;
3. Install auxiliary cables to increase the longitudinal direction stiffness of the mid-pylon to improve the global stiffness, and structural performance of the structure;
4. Crossed cables are used for anchoring in the middle of the main span to form a truss system to improve the global stiffness and performance of the structure.

However, increasing the height of the main girder to improve the global stiffness of the structure is more suitable for multi-pylon cable-stayed bridges with smaller spans. For example, the French Millau Bridge adopts this method. Nevertheless, it is uneconomical for a multi-pylon cable-stayed bridge with large spans like JYRB. Installing the auxiliary cables between the pylons can play a role in multi-pylon cable-stayed bridges with medium and small spans. Well known, the sag significantly increases with the increase of the cable length, thereby reducing the global stiffness and cable stability^11^. Therefore, this approach has a minimal impact on long-span multi-pylon cable-stayed bridges. For cable-stayed bridges with a main span of up to $600\,\textrm{m}$, the method of crossed cables can indeed improve the global stiffness, but the mutual influence of the wind-induced vibration of the stay cables at the cable intersections and how to effectively suppress them are issues that need further research. Therefore, this project adopts the method of increasing the stiffness of the pylon to enhance the global stiffness. For conventional in-line pylons in the longitudinal direction, the global stiffness of the middle pylon should be increased if the longitudinal width of the middle pylon increases. Suppose the global stiffness meets the requirements of the specification when the conventional in-line pylon in the longitudinal direction is adopted. In that case, the width of the middle pylon along the longitudinal direction should be more than $16\,\textrm{m}$ according to the calculation results of the midas model, resulting in poor economic efficiency. Therefore, this method needs to be improved. However, the longitudinal opening of the pylon column can significantly enhance the longitudinal stiffness of the middle pylon. This should be a more economical and reasonable plan.

Theoretically, the longitudinal A-shaped pylon is the most effective structure for the pylon of longitudinal opening. However, it may increase the foundation size along the longitudinal direction and the water-blocking surfaces. The lower part below the bridge deck of the pylon is retracted to form a diamond-shaped pylon, which can reduce the amount of engineering of the pylon and the foundation while ensuring the global stiffness of the pylon.

If the pylon is opened longitudinally into a diamond shape, and the double-column pylon is still used transversely, the pylon will have four legs above the bridge deck. This will result in complex forces for this pylon structure. In addition, the stay cables and the four-leg pylon coexist in one space, resulting in complicated spatial lines and poor visual effects. It is necessary to use a single-pillar pylon in the transverse direction. Therefore, the overall appearance will be concise and smooth, producing a unique visual effect.

For the single-column pylon that occupies the central space of the main beam, optimizing the lateral structure size of the pylon under the premise of meeting various structural and technical requirements is the foothold to reduce the scale of the project and reduce the project investment. It is suitable for the construction conditions of JYRB and meets the structural force requirements of a cable-stayed bridge with a main span of $600\,\textrm{m}$. If concrete pylons are used for JYRB, the minimum lateral dimension of the pylon will be $6.6\,\textrm{m}$. To further optimize the lateral dimensions of the pylon structure, the following efforts should be performed: material selection and structural composition. Although steel pylons have been used in China with mature design and construction experience. In addition, the steel structure pylon has excellent bearing capacity, seismic performance, and structural durability. Its smooth and clean outer surface makes obtaining a better aesthetic effect easier. However, constructing steel structure pylons requires large machining and lifting equipment, which may increase the project cost. It is worth noting that for a single pylon, such as a diamond-shaped pylon in the longitudinal direction, the flexural stiffness and compressive stiffness of steel structure pylons are disadvantageous if the geometric dimensions of the steel structure pylon and the concrete pylon are the same. Therefore, combining the advantages and disadvantages of the concrete pylon and steel pylon, steel-concrete composite pylons may be a good choice.

**Figure 5:**
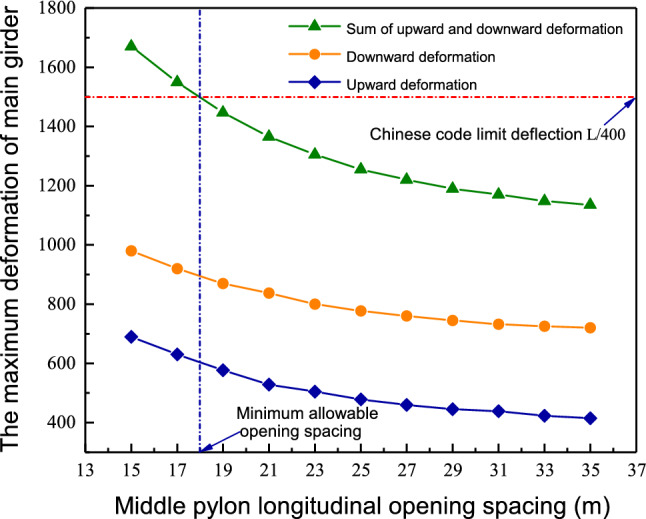
The longitudinal opening spacing of the middle pylon.

Since the longitudinal opening spacing of the pylon will affect the global stiffness of the entire bridge when the longitudinal opening spacing is too small, the global stiffness of the entire bridge may not be enough. When the longitudinal opening spacing is too large, it will be uneconomical. Therefore, a rod system finite element model of the entire bridge was established to determine a reasonable longitudinal opening spacing. Moreover, the longitudinal opening spacing of the middle pylons was used as a parameter to determine the impact of the longitudinal opening spacing on deformations of the main girder under live load, to determine the impact of the middle pylon longitudinal opening spacing on the global stiffness of the bridge. Figure 5 shows the maximum deformation of the main girder under life loads with different middle pylon longitudinal opening spacings. As seen from Fig. 5, when the longitudinal opening spacing of the middle pylon is less than 18 m, the maximum deformation value of the main girder under life loads will exceed the Chinese code limit deflection L/400. This shows that if a longitudinal diamond-shaped single pylon is used for a cable-stayed bridge with a main span of 600 m, the longitudinal opening spacing cannot be less than 18 m to meet the global stiffness requirements of the bridge. Considering the stiffness, aesthetics, economy and convenience of construction, the final longitudinal opening spacing of the bridge is set at 21 m.

## Steel-concrete-steel sandwich composite pylon section stiffness analysis

**Figure 6:**
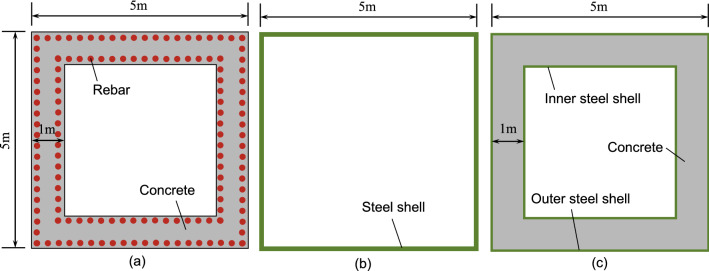
Three types of cross-sectional diagrams: (**a**) concrete section; (**b**) steel section; (**c**) steel-concrete-steel sandwich composite section.

The pylon is an eccentric bending member, while the axial stiffness $\textrm{EA}$ and the flexural stiffness $\textrm{EI}$ are two important indexes to measure the pylon’s ability to resist the external force deformation. Therefore, the basic model is abstracted based on the actual pylons to compare the stiffness performance of steel, concrete pylons, and composite pylons. For comparison, a box section with an outer profile of $5\,\textrm{m}\times 5\,\textrm{m}$ is selected. The wall thickness of the concrete pylon and the composite pylon is $1\,\textrm{m}$, and the wall thickness of the steel pylon is calculated based on the equivalent cost. As shown in Fig. 6, there are a total of 332 rebars in the section of the concrete pylon, which are arranged in two layers. The spacing of the rebars is $100\,\textrm{mm}$, the diameter of the steel bars is $36\,\textrm{mm}$, and the thickness of the concrete cover depth is $25\,\textrm{mm}$.They kept the composite and concrete pylon sections at the same steel content to ensure the material costs were the same. Regarding the pylon of JXZB, the outer steel shell thickness could be $14\,\textrm{mm}$, and the inner steel shell thickness could be $6\,\textrm{mm}$ through conversion. To keep the material cost of the steel pylon consistent with that of the concrete pylon, it is calculated based on $6000\,\textrm{yuan}/\textrm{ton}$ for steel and $500\,\textrm{yuan}/\textrm{square}$ for concrete, and the converted steel shell thickness is $25.26\,\textrm{mm}$.

For the above three cross-sections of the pylon, the comparison of the axial compression stiffness $\textrm{EA}$ and the bending stiffness $\textrm{EI}$ are shown in Table 2. The section stiffness calculation results come from the section calculator of the finite element software Midas.

**Table 2:** Comparison of section stiffness of different types of pylons.

Type	Axial compression stiffness EA(*N*)	Bending stiffness EI($N{\cdot }mm^{2}$)
Concrete section	$6.25\times 10^{17}$	$1.82\times 10^{24}$
Steel section	$1.14\times 10^{17}$	$4.76\times 10^{23}$
Steel-concrete-steel sandwich composite section	$6.25\times 10^{17}$	$1.83\times 10^{24}$

It can be seen from the table that the axial stiffness of the composite pylon section is the same as that of the concrete pylon section, and the flexural stiffness of the composite pylon section is slightly larger than that of the concrete pylon section. Both composite and concrete pylon sections have significantly more section stiffness compared to steel pylon sections. Therefore, the composite pylon section inherits the advantages of the significant rigidity of the concrete pylon sections. At the same time, the steel shells in the composite structure pylon are used as a part of the stressed structures, which can replace part of the rebars to reduce the number of rebars and reduce the difficulty of construction. The steel shells can also be used as a template to improve the convenience and efficiency of construction. In summary, the composite pylon has advantages in mechanical properties and construction compared with steel and concrete pylons.

### Brief mechanical performance analysis

**Figure 7:**
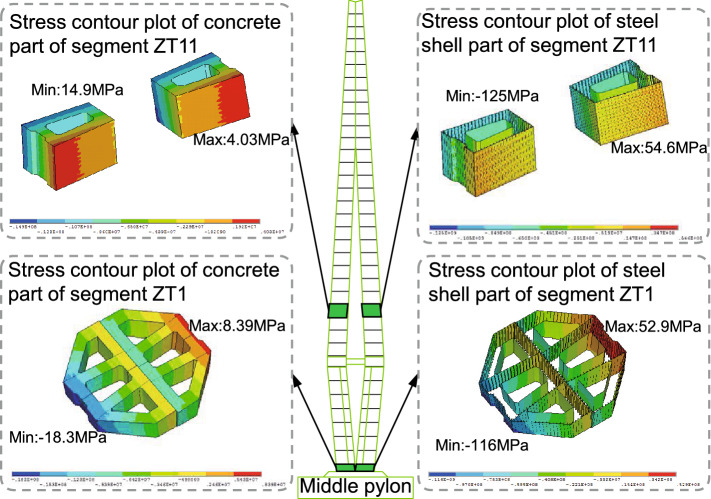
The most unfavorable calculation result of the pylon under the service limit state.

**Figure 8:**
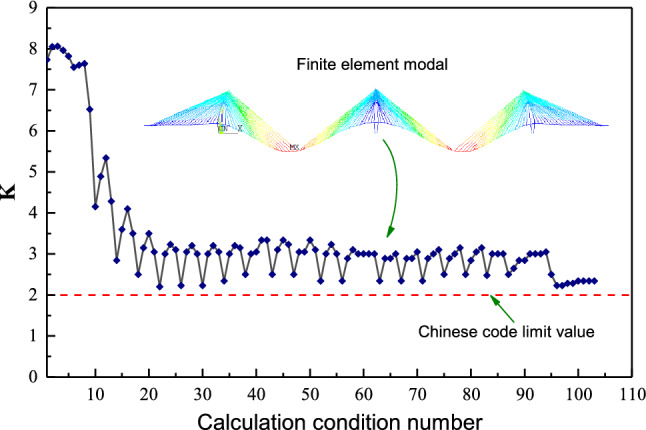
The longitudinal opening spacing of the middle pylon.

The finite element model of JXZB was established via Midas Civil to simulate the process of the construction stages to determine the most unfavourable section position and the most unfavourable working condition. Subsequently, the pylon segment local analysis model using solid elements was established via the ANSYS. The maximum tensile stress of concrete is $8.39\,\textrm{MPa}$, and the maximum compressive stress is $-18.3\,\textrm{MPa}$, which appears at the ZT1 segment. The maximum tensile stress of the steel shell is $54.6\,\textrm{MPa}$, and the maximum compressive stress is $-125\,\textrm{MPa}$, which appears at the ZT11 segment, see Fig. 7. Although the maximum tensile stress of the concrete in the steel shell exceeds the design value of the tensile strength of C50 concrete, the calculated maximum crack width is $0.087\,\textrm{mm}$, which is less than the limit value in the Chinese code. Therefore, the steel-concrete-steel sandwich composite pylon’s strength meets the Chinese code’s requirements. In addition, Mi- das software also calculated the structural nonlinear stability safety factors of 103 calculating cases in the construction and operation stages. The calculation results are shown in Fig. 8. It can be seen from the figure that with the development of the construction progresses, the nonlinear stability coefficient K gradually becomes smaller. After the completion of the bridge, the value tends to be stable and is always more significant than the specification limit. Perhaps some people are more concerned about the shear resistance of the PBL shear key and the void of concrete. Other research teams have conducted experimental studies on the horizontal and vertical PBL shear keys of JXZB, and the results show that the horizontal and vertical PBL shear keys of JXZB all have good shear resistance. For the void rate of concrete, our research team is also testing it in a full-scale model test. For specific results, please see the content in the next section.

## Full scale model process test

The steel-concrete-steel sandwich composite pylon is now the first application in a large-span bridge. Due to its unique complex structure, it is not easy to manufacture, install and construct. To verify the feasibility and adaptability of the construction process, a full-scale model process test of this pylon was carried out, focusing on the steel shell hoisting and positioning, the on-site reinforcement connection, concrete pouring process, the work performance of the concrete and the law of temperature and strain changes. Through the full-scale model process test, the construction processes of one steel shell segment of the pylon were previewed, the problems in the construction processes were found, and the matters needing attention were put forward to guide the formal construction of the pylon.

### Steel-concrete-steel sandwich composite pylon construction process

Based on the previous construction experience, it is determined that the construction process of the steel-concrete-steel sandwich composite pylon is as follows:

Step 1: Manufacture and pre-assemble the steel shell segments in the factory;
Step 2: Transport the steel shell segments;
Step 3: Hoist the steel shell segments;
Step 4: Assemble the steel shell segments;
Step 5: Connect rebars in the steel shell and weld the steel shell segments;
Step 6: Pour and vibrate the concrete in the steel shell;
Step 7: Cure the concrete in the steel shell;
Step 8: Chisel the top of the concrete and remove the slag;
Step 9: Hoist the next segment of the steel shell.

### Test process and data monitoring

**Figure 9:**
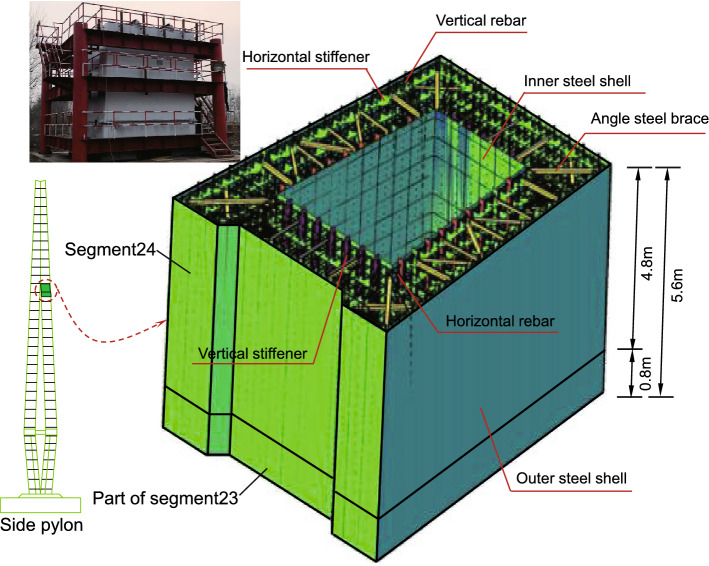
Full scale segment model.

**Figure 10:**
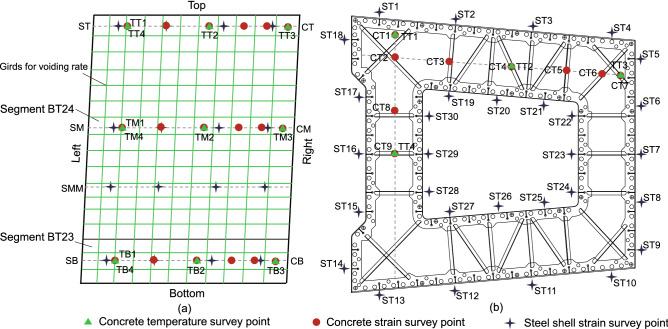
Survey points layout of the full scale model: (**a**) distribution of survey points in elevation map; (**b**) distribution of survey points in pylon section.

1. Test segments selection
  The selection of test segments needs to reflect the structural characteristics of each segment of the pylons and possible construction difficulties, and it also needs to reflect the key points of quality control of the pylon segment construction at each stage. Combining with the economy, after careful consideration, the segment BT24 (single limb) and $800\,\textrm{mm}$ of the upper part of the segment BT23 (connected to the segment BT24) of the side pylon were chosen as the full-scale test model segments. The segment BT24 is in the uppermost segment of the middle parts of the side pylon, as shown in Fig. 2g. The test model has a height of $5.6\,\textrm{m}$ and a weight of $30.5\,\textrm{t}$. The full-scale model structure of the test segment is shown in Fig. 9.
2. Test processes
  The steel shell segments are manufactured and assembled in the factory. Then, they are transported to the bridge site after passing the inspection and acceptance. The steel shell segment BT23 is hoisted in place by a car crane. Then, it is fixed steel shell segment BT23 on the foundation. The bottom $30\,\textrm{cm}$ high concrete is pre-poured, and the remaining $50\,\textrm{cm}$ high concrete is poured together with the steel shell segment BT24. After the pre-poured concrete reaches the design strength, the steel shell segment BT24 is hoisted, and the initial positioning is completed. Temporarily fix the steel shell segments with temporary matching parts between the segments. The temporary matching parts are released after the welding connection between the steel shell segments is completed. Symmetric welding is used for segment connection to avoid excessive temperature gradient of the steel shell due to asymmetric welding. After the welds are qualified, the vertical rebars will be connected. When the steel shell segments are connected, the concrete will be poured layer by layer. The thickness of each layer of concrete is about $40\,\textrm{cm}$. Water storage and maintenance are carried out when the concrete is initially set.
3. Data monitoring
  Due to the thin steel plate, it is necessary to monitor the deformation of the steel plate during the whole process. Strain sensors are arranged on the surface of the steel plate to get the deformations of the hoisting, welding and concrete pouring process, as shown in Fig. 10. At the same time, to ensure the quality of shrinkage-compensating concrete, the temperature, and deformation of the concrete were also monitored in the test. The strain and the temperature are collected by Changsha Jinma JMBV-1116 multi-functional data collector. The displacement data is collected by electronic dial gauge. The layout of the corresponding measurement points is shown in Fig. 10. After the concrete curing is completed, ultrasonic testing is used to detect the hollowing rate of the steel shell.

### Test results and analysis

1. Deformation of steel shell

**Figure 11:**
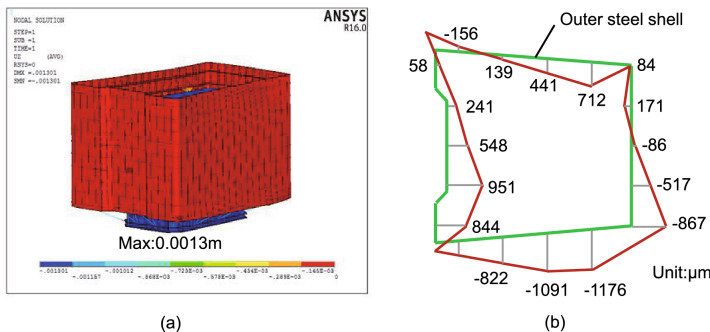
The deformation of steel shell: (**a**) the finite element analysis results of hoisting; (**b**) the welding deformation of the top section.

During the hoisting process, four hoisting points are set on the outer wall of the outer steel shell of the steel shell segment so that the maximum relative deformation may occur in the inner steel shell. The finite element analysis results of hoisting show that the maximum displacement occurs on the inner steel shell, the displacement value is $1.3\,\textrm{mm}$, see Fig. 11a. The actual measurement results during hoisting show that the maximum displacement of the steel shell occurs on the inner steel shell during the hoisting process, and the displacement value is $1.1\,\textrm{mm}$, which is consistent with the finite element analysis results.

The uneven temperature field of the welding process is prone to residual welding deformation. Suppose the welding deformation value is considerable, which will affect the installation and reliability of the structure. Therefore, it is necessary to pay attention to the residual welding deformation. According to the actual measurement results, the welding deformation of the top section of the steel shell segment is shown in Fig. 11b. The maximum deformation during the welding process is about $1.2\,\textrm{mm}$. It shows that the welding process has negligible influence on the deformation of the steel shell.

The deformation values of the steel shell are measured to obtain the influence of the lateral pressure and the heat of hydration reaction on the deformation of the steel shell when the concrete is poured. Table 3 shows the maximum deformation values of the outer layer steel shell and the inner layer steel shell of the typical sections (top section, middle section, and bottom section) of the segment. It can be seen from Table 3 that the deformation of the outer steel shell is greater than that of the inner steel shell, and the deformation of the top section is like that of the bottom section, but both are greater than the deformation of the middle section. This may be related to the inclination of the steel shell segment. In general, the deformation level of the segment is relatively low, and the maximum deformation value is smaller than $1.5\,\textrm{mm}$. Therefore, the concrete pouring process has little effect on the deformation of the steel shell.

**Table 3:** Maximum deformation of steel shell during concrete pouring (Unit:$\mu$ $m$).

Location	Maximum deformation of inner layer steel shell	Maximum deformation of outer layer steel shell
Top section	297	1404
Middle section	0	974
Bottom section	304	1266

(2) The temperature of concrete

As the experiment was carried out in the field in winter, the temperature was about 0 to $5.0\,^{\circ }\textrm{C}$. Therefore, the concrete is mixed with warm water, and the temperature of the mixing water is $16.8\sim 17.5\,^{\circ }\textrm{C}$. The temperature of the concrete leaving the mixing station is about $8.5\,^{\circ }\textrm{C}$, and the pumping temperature of concrete is about $10.0\,^{\circ }\textrm{C}$. The temperature monitoring results of the concrete core showed that:

1. After the concrete was poured, the internal temperature of the concrete began to rise, the highest temperature was $47.2\,^{\circ }\textrm{C}$, the actual maximum temperature rise of the concrete was about $37.0\,^{\circ }\textrm{C}$, and the temperature peak appeared at $43\textrm{h}$ after the start of concrete pouring;
2. The maximum temperature difference between the concrete inside and surface occurred 49h after pouring, and the temperature difference was $27.1\,^{\circ }\textrm{C}$;
3. The internal temperature of the concrete is the same as the ambient temperature of $193\,\textrm{h}$ after the start of pouring, and the internal temperature dissipation is completed.

**Figure 12:**
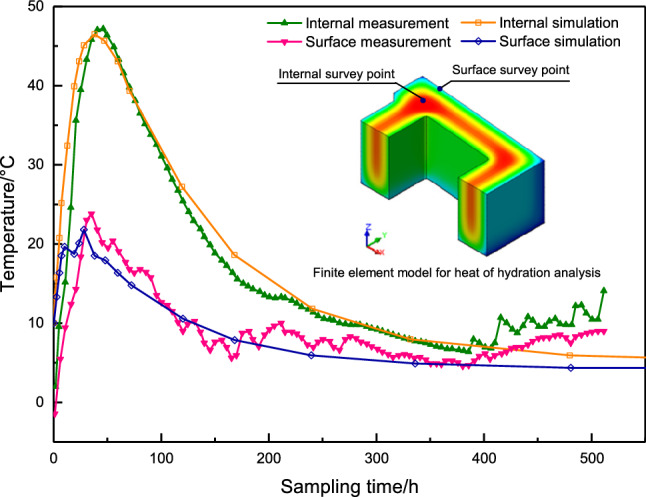
Concrete temperature time history curve.

The finite element model of the hydration heat analysis of the experimental model was established by using solid elements in finite element software. According to the simulation temperature calculation results, the hydration heat time history analysis is carried out and compared with the actual measured values on-the-spot. The time history curve of concrete temperature is shown in Fig. 12. It can be seen from Fig. 12 that the calculated maximum temperature of the model concrete is $46.7\,^{\circ }\textrm{C}$, which is a little different from the actual measured value via the temperature sensor. The calculated peak temperature and the appearance time of the concrete temperature peak are consistent with the measured value. The law of concrete temperature rises and drops is consistent with the measured values, which shows that the calculation result is accurate and reliable. For the high temperature in the summer case (the ambient temperature is $28.0\,^{\circ }\textrm{C}$), the calculation results show that the maximum temperature rise in the concrete is $67.8\,^{\circ }\textrm{C}$, the temperature peak time appears $44\,\textrm{h}$ after the start of pouring, and the temperature rise is about $39.8\,^{\circ }\textrm{C}$. Therefore, measures such as lowering the pouring temperature, controlling the temperature and humidity environment of the on-site pouring area, and choosing the appropriate pouring time should be taken in the hot season to ensure that the internal temperature of the concrete meets the design requirements.

(3) The deformation of concrete

**Figure 13:**
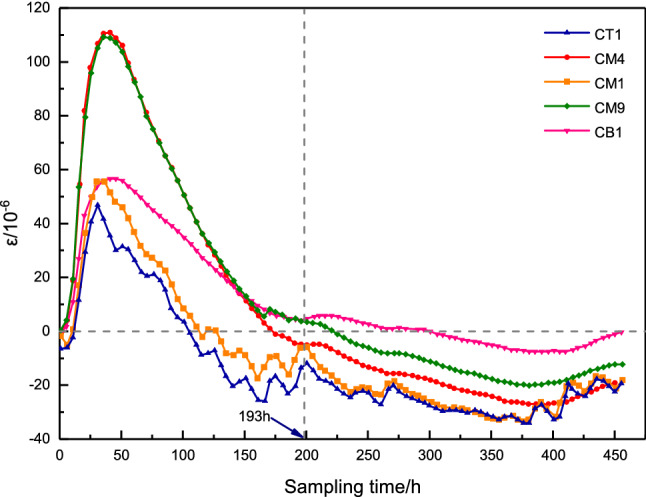
Concrete strain time history curve.

The effect of concrete deformation control can be verified according to the strain of concrete in different parts. The collected concrete strain field measurement data was organized and drawn into concrete strain time history curves, as shown in Fig. 13. It can be seen from Fig. 13 that the maximum strain value observed inside the concrete is about $111\times 10^{-6}$, and the minimum strain value is about $-35\times 10^{-6}$. The strain change history is consistent with the concrete temperature change history. The appearance time of the maximum strain value is the same as the appearance time of the maximum temperature value, and the internal temperature rise of the concrete has a more significant influence on the concrete deformation. After the heat in the concrete dissipates ($193\,\textrm{h}$), the deformation value of each measuring point is stable, and the strain value of different measuring points is stable at $5.8\times 10^{-6}{\sim }-35\times 10^{-6}$. The deformation of the concrete changes slowly with the fluctuation of the ambient temperature.

(4) The hollowing rate of the steel shell

**Figure 14:**
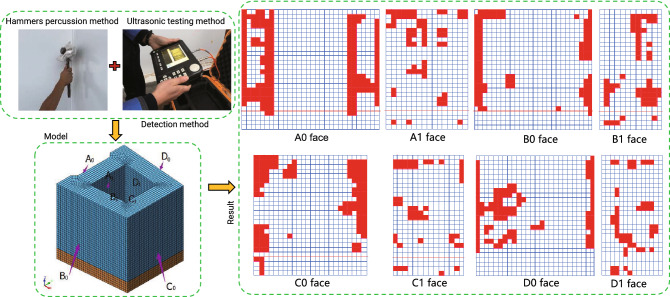
Detection results of concrete hollowing.

A $40\times 40\,\textrm{cm}$ square is used as the control grid, and each grid is a common measurement area. Each surface of the steel shell is divided into standard measurement areas. If there are concrete voids in the $40\times 40\,\textrm{cm}$ square standard measuring area, the grid will be refined in the measuring area, and the size of the refined grid will be $10\times 10\,\textrm{cm}$. The void area with less than 0.5 refined grids is discarded, and the void area with more than 0.5 refined grids is counted as 1 refined grid. Figure 14 shows the distribution of void areas based on the measured results. It can be seen from the void distribution figure that the void rate of the upper part of the steel shell is significantly higher than that of the lower part, which may be caused by the floating and pooling of internal air bubbles during the concrete pouring process. The void rate on both sides of the outer steel shell is higher than that in the middle, while the void rate in the middle of the inner steel shell is higher than that on both sides. The statistical data is given in Table 4. The void rate of the outer steel shell is about 10–15%, and the void rate of the inner steel shell is about 6–11%. The total void rate of the outer steel shell is 12.41%, and the total void rate of the inner steel shell is 7.86%. This means that the void rate of the inner steel shell is significantly lower than that of the outer steel shell. Therefore, it is necessary to improve the pouring method further and curing conditions in the actual bridge construction to reduce the void rate, such as strengthening the vibrating of the possible void areas or the built-in exhaust pipe and at the same time adopting some insulation measures during the curing of the concrete to reduce the temperature difference of the steel shell and concrete. For areas with serious voids, secondary grouting may be required for remediation.

**Table 4:** Void data statistics table (Unit:$\textrm{cm}^{2}$).

Location	Surface	Area	Void area	Void rate (%)	Total area	Total void area	Total void rate (%)
Outer steel shell	A0	312000	47775	15.30	1014000	125800	12.41
	B0	223600	22500	10.06			
	C0	254800	32000	12.56			
	D0	223600	23525	10.52			
Inner steel shell	A1	160000	10850	6.78	525000	41275	7.86
	B1	115000	9325	8.11			
	C1	135000	8250	6.11			
	D1	115000	12850	11.17			

## Key technology of pylon construction

The steel-concrete-steel sandwich composite pylon is a new type of pylon that combines permanent and temporary structures. The steel shell is not only involved in structural force but also a concrete pouring formwork. The rebars in the pylon are positioned and assembled with the steel shell in the factory, which eliminates the process of assembling reinforcement on-site, thus realizing the rapid construction of the pylon. According to the height and weight of the pylon segment, the steel shell in the lower part is hoisted by a floating crane and the steel shell in the middle and upper part is hoisted by a pylon crane. The lower part installation uses the temporary construction support of the 0# block of the main girder as the construction work platform, and the middle and upper parts installation uses a hydraulic automatic climbing operating platform system.

### Accurate construction of the first segment

**Figure 15:**
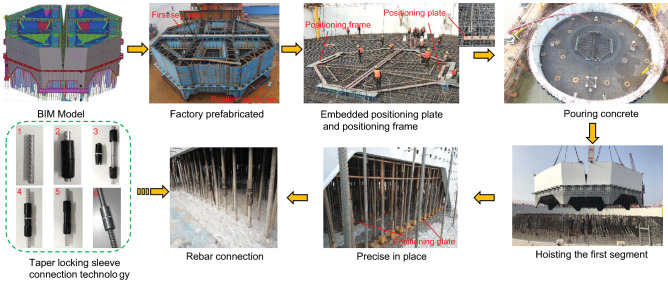
Precise positioning technology for the first segment.

The precise positioning and installation of the first segment of the steel shell is the foundation to ensure the construction of the pylon, and its installation accuracy directly affects the installation accuracy of the entire pylon. Since the steel shell segments have been processed in the factory, the in-site installation is only a reproduction of the assembly in the factory, and its spatial position is not adjustable segment by segment like a concrete pylon. Therefore, the space for the first pylon segment’s bottom plate and top surface must be very accurate. JYRB uses the following aspects to ensure the accurate positioning of the pylon (see Fig. 15):

1. Using BIM (Building Information Modeling) technology to analyze the collision and positioning of rebars. A BIM model was established, including the first segment of the pylon with positioning embedded parts, the platform, and the pylon base, and conducting collision inspection. Accurately locate the specific positions of each vertical embedded rebar through the BIM model to realize the precise alignment of the embedded vertical main bar on the top surface of the platform and the vertical main bar pre-installed in the steel shell.
2. Two rebar positioning plates are installed in the platform to improve the precision of embedded rebars. The rebar holes on the positioning plates are accurately positioned and drilled by CNC (Computer Numerical Control) machine tools.
3. Embedding positioning frames to ensure the installation accuracy of the steel shell of the first segment. Vertical and horizontal positioning frames are set at the foot of the steel shell of the first segment in the factory. At the same time, another positioning frame is embedded during the construction of the platform to ensure the installation accuracy of the first segment of the steel shell.
4. Using taper locking sleeve connection technology. It is difficult to control and match the interface between the segments because of the large-size profiled cross-section. Factors such as internal and external wall stiffeners, angle steel, horizontal steel bars, and stirrups crisscross, as well as unpredictable deformations and the impact of concrete pouring, all affect the installation accuracy of the first segment of the steel shell. Thence, it is difficult to use straight threaded sleeves to connect the vertical rebars. Taper locking sleeves connect the vertical rebars in the construction process, which solves the connection problem of pre-embedded rebars in large section segments.

### Steel shell segment hoisting and precise positioning

**Figure 16:**
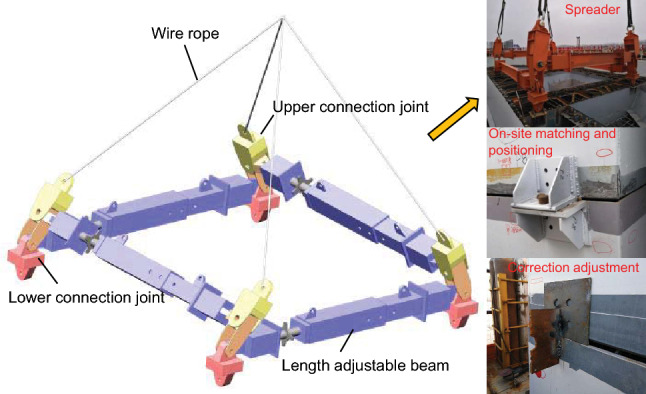
Steel shell segment hoisting and precise positioning.

Because the segments of the steel shell are special-shaped structures, and the centre of the segment does not overlap in the vertical projection, it brings more significant difficulties to the precise positioning of the segment hoisting. For this reason, a new type of adjustable precision strut spreader was developed for segmental hoisting. As shown in Fig. 16, the spreader consists of 4 beams, four pairs of upper and lower connection joints, and steel wire ropes. By adjusting the lengths of the spreader beams and setting the adjustment shackle at the end of the steel wire ropes, the function of adjusting the distance of the lifting point, the inclination of the segment, and the lifting centre of gravity are realized. It solves the hoisting problem of the special-shaped steel shell segments of the pylon.

To quickly locate and connect the segment to the installed segment, matching parts are installed between those segments in the factory. After the inter-segment connection is completed, the matching device is released. When performing precise position adjustment on-site, first adjust the supporting steel plates of the eight matching parts at the bottom of the steel shell segment according to the matching data in the factory and monitoring instructions. Secondly, each pylon limb retains one matching part locking point punching nail and removing the remaining three matching parts punch nails and bolts so that the segment can be slightly rotated in the horizontal plane. At the same time, the matching parts are kept fit, and the side is filled with steel plates for torsion correction. After the adjustment is in place, it needs to be rechecked, and the subsequent process can be constructed after the requirements are met.

It is worth pointing out that the steel shell segment has not been machined on the end face. Compared with the steel structure pylon with the machined end face, the unmachined steel shell segments exhibit a low vertical baseline accuracy and pre-assembly accuracy, the poor fixing effect of matching parts. In this respect, the steel-concrete-steel sandwich composite pylon will be more prone to errors than steel pylons. Therefore, it is necessary to set more adjustment segments to correct and adjust the error.

### R &D and application of pylon construction platform

The hydraulic automatic climbing platform is widely used in building high pylons in bridge engineering. The conventional climbing system includes two parts: tracks and a climbing platform. In the climbing construction, the track is usually climbed first and followed by the construction platform. The construction process of the whole system is complicated, and the construction period is extended. The tracks and construction platform adopts a different design, increasing the entire system’s space size and weight.

**Figure 17:**
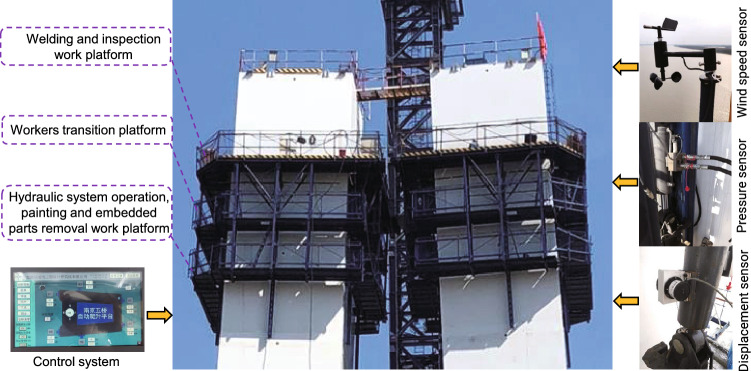
Hydraulic automatic climbing operating platform.

A new hydraulic automatic climbing platform is used to construct steel-concrete-steel sandwich composite bridge pylons. This new automatic climbing platform includes three layers to provide an operating platform for steel shell hoisting, inter-segment welding and surface coating. At the same time, the system also introduces a set of intelligent control systems, i.e. standard small hydraulic systems, real-time monitoring systems, and intelligent early warning systems, see Fig. 17. Compared with the traditional climbing platform, this automatic climbing platform realizes the synchronous climbing of the track and the construction platform, reducing the operation steps and the overall weight of the climbing device. At the same time, the manual operating platform is scalable, which avoids repeated manual disassembly and assembly work, thereby improving work efficiency and the wind resistance of the construction platform.

### Concrete pouring and curing in steel shell

An automobile pump pours the concrete of the lower part of the pylon. Due to the limitation of the pumping height of the automobile pump, the concrete of the middle and upper parts is conveyed by a large pylon crane hoisting the hopper. The slump of the concrete can be controlled at $18\sim 20\,\textrm{cm}$, ensuring the concrete construction performance. Cold storage is used to cool the sand and stone in the hot summer weather. At the same time, the crushed ice mechanism is used to cool the mixing water to control the concrete entering the steel shells to ensure that the core temperature of the concrete is not greater than $65.0\,^{\circ }\textrm{C}$. Vibration holes are reserved on the horizontal stiffening plate in the steel shell. During the concrete pouring and vibrating process, the vibrating rod is inserted into the vibrating hole to ensure the compactness of the concrete. When pouring the concrete close to the steel shell siding, the $70\,\textrm{mm}$ diameter concrete vibrating and vent holes opened on the circumferential stiffening plates are fully utilized to ensure the quality of the concrete pouring construction.

**Figure 18:**
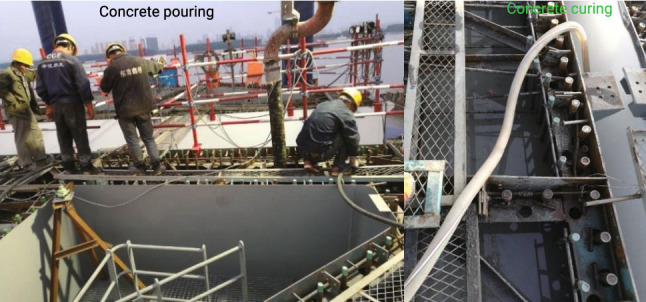
Concrete pouring and curing in steel shell.

To avoid cracks between the concrete and the steel shell due to the rapid loss of water on the surface of the concrete, the top concrete should be protected by water storage in time after the initial set of the concrete, and the water storage depth should not be less than $10\,\textrm{mm}$. Forty-eight hours after the final set of the concrete, the top surface is chipped with an electric pick, and the depth is controlled to be about $10\,\textrm{mm}$. The concrete slag, after chiselling, is cleaned with a vacuum cleaner to improve the efficiency and effect of slag removal. The pouring and curing of concrete are shown in Fig. 18.

### Comparative analysis

The steel-concrete-steel sandwich composite pylons have been applied for the first time in such long-span cable-stayed bridges. It has achieved ideal results in increasing the rate of factory production, rapid construction, and reducing labor input. The comparison with concrete pylons and steel pylons in terms of construction investment and construction efficiency is shown in Table 5.

**Table 5:** Comparison table of concrete pylons, steel pylons and steel-concrete-steel sandwich composite pylons.

Items	Concrete pylons	Steel pylons	Steel-concrete-steel sandwich composite pylons
Structural features	Large cross-section size, large amount of rebars and concrete	Large cross-section size, large amount of steel	Small cross-section size, less rebars
Construction features	Rebar binding, formwork installation, concrete pouring construction on-site	Factory manufacturing, assembly installation with large and heavy lifting equipment	Factory manufacturing, assembly installation with light lifting equipment, concrete pouring construction on-site
Working efficiency	7 days/segment	2 days/segment	4 days/segment
Labor input	about 60 people	about 10 people	about 15 people
Security	Work intensity is high, low personnel safety	Mostly mechanized operations, high personnel safety	Mostly mechanized operations, high personnel safety
Construction period	About 19 months	About 8 months	About 14 months
Cost ratio	1	2.9	1.2

It can be seen from Table 5 that there are few temporary facilities for the construction of steel-concrete-steel sandwich composite pylons, the construction speed is about 1.4 times that of concrete pylons, and the labour input is only about 1/4 of that of concrete pylons. Most of the work is done in the factory, which can significantly increase the factory-based rate of pylon construction and guarantee the construction quality. Due to the reduction of labour, safety management and control are easy to implement, and the safety of the construction personnel is improved. Although steel pylons may be more prominent than steel-concrete-steel sandwich composite pylons in these respects, the construction costs of steel pylons are about three times that of concrete pylons due to the ample lifting equipment required for steel pylons. However, the steel-concrete-steel sandwich composite pylons cost only 1.2 times that of the concrete pylons. Therefore, it spends a small amount more than the concrete pylon and obtains better results in many other aspects. Steel-concrete-steel sandwich composite pylon may be a good choice for large span cable-supported bridges.

## Conclusions

The steel-concrete-steel sandwich composite pylon is a new type of steel-concrete composite pylon that was first applied to the long-span cable-stayed bridge. This study focuses on the design ideas and key construction techniques of this type of pylon and draws the following conclusions:

1. The calculation results show that the bridge pylons exhibit good mechanical properties and can ensure the structure’s safety.
2. The full-scale process model test confirmed the feasibility of rapid construction of the structure and provided early warning of possible problems in actual constructions.
3. The application of BIM technology, the research and development of special spreaders and construction platforms ensure the precise installation of structures.
4. The factory-manufactured modular assembly of the reinforced steel shell structure effectively reduces the intensity and difficulty of on-site operations, improves the quality of the project and reduces the construction risk.
5. The successful application of this bridge pylon marks the formation of a complete set of construction technology of steel-concrete-steel sandwich composite pylon, which can be widely used in similar bridges.

## Data Availability

Some or all data, models, or codes that support the findings of this study are available from the corresponding author upon reasonable request.
